# Impact on survival of estrogen receptor, progesterone receptor and Ki-67 expression discordance pre- and post-neoadjuvant chemotherapy in breast cancer

**DOI:** 10.1371/journal.pone.0231895

**Published:** 2020-04-16

**Authors:** Yuqin Ding, Kaijing Ding, Hongdan Qian, Xingfei Yu, Dehong Zou, Hongjian Yang, Wenju Mo, Xiangming He, Fanrong Zhang, Chengdong Qin, Yurong Zheng, Xiaowen Ding

**Affiliations:** 1 Department of Breast Surgery, Institute of Cancer Research and Basic Medical Sciences of Chinese Academy of Sciences, Cancer Hospital of University of Chinese Academy of Sciences, Zhejiang Cancer Hospital, Hangzhou, Zhejiang, China; 2 Department of Child Psychology, Zhejiang University Affiliated Mental Health Center, Hangzhou Seventh People's Hospital, Hangzhou, Zhejiang, China; 3 Department of General Surgery, Deqing County Hospital of Traditional Chinese Medicine, Huzhou, Zhejiang, China; American Society for Investigative Pathology, UNITED STATES

## Abstract

**Purpose:**

To investigate whether estrogen receptor (ER), progesterone receptor (PR) and Ki-67 expression discordance before and after neoadjuvant chemotherapy (NAC) correlates with prognosis and treatment of breast cancer patients.

**Methods:**

The study cohort included 482 breast cancer patients at the Zhejiang Cancer Hospital from January 1, 2008, to December 31, 2018. Core needle biopsies and excised tissue biopsies pre- and post-NAC were obtained. Immunohistochemistry was used to determine ER, PR and Ki-67 status. The relationship between biomarker discordance before and after NAC and clinicopathological features was compared retrospectively.

**Results:**

ER (n = 482), PR (n = 482) and Ki-67 (n = 448) expression was assessed in the same lesion pre- and post-NAC. Discordance in the three markers pre- and post-NAC was observed in 50 (10.4%), 82 (17.0%) and 373 (77.4%) cases, respectively. Positive-to-negative PR expression changes were the most common type of discordance observed. The risk of death in patients with a PR positive-to-negative conversion was 6.58 times greater than for patients with stable PR expression. The risk of death in patients with increased Ki-67 expression following NAC treatment was 2.05 times greater than for patients with stable Ki-67 expression.

**Conclusion:**

Breast cancer patients showed changes in ER, PR and/or Ki-67 status throughout NAC, and these changes possibly influenced disease-free survival and overall survival. A switch to negative hormone receptor expression with increased Ki-67 expression following NAC could be indicators of a worse prognosis. Biomarker expression investigations following NAC may potentially improve patient management and survival.

## Introduction

Although neoadjuvant chemotherapy (NAC) is increasingly used for breast cancer treatment, neoadjuvant endocrine therapy is also administered based on the presence of biomarkers such as estrogen receptor (ER), progesterone receptor (PR) and Ki-67 [1]. At least twenty percent of women with early-stage breast cancer will later develop metastatic disease [2, 3]. Endocrine therapy often provides a benefit to patients with ER-positive and/or PR-positive hormone-dependent breast cancer [4, 5]. Therefore, hormone receptor (HR) detection assays, which measure ER and PR, have become standard practice for endocrine treatment [6]. Several small studies have revealed a lack of stability of HR and/or Ki-67 biomarker expression during tumor progression in breast cancer [4, 6, 7]. It is currently unknown how NAC modulates these biomarkers. If therapy-predictive biomarkers change throughout NAC, investigating biomarker expression in lesions before and after NAC could provide additional important information that could improve patient treatment management.

Little is known about the predictive or prognostic value of altered receptor status. Several investigators attempted to correlate receptor changes to treatment response, but conflicting conclusions were drawn [5]. Retrospective analyses of primary and recurrent breast cancers suggest that receptor expression discordance not only is statistically significant, but also can be associated with poorer survival [8]. This decrease in survival could perhaps be a result of the use of inappropriate targeted therapy or the outgrowth of tumors with a more unstable phenotype and therefore more aggressive phenotype. Other prospective studies that include a high proportion of women with operable disease have not evaluated the effects of HR expression discordance throughout therapy on patient survival [6].

In this retrospective study, we evaluated HR and Ki-67 expression before and after NAC in a cohort of patients from Zhejiang, China. We hypothesized that discordance in biomarker expression would be correlated with a statistically significant difference in the prognosis of breast cancer patients.

## Patients and methods

**Fig 1:**
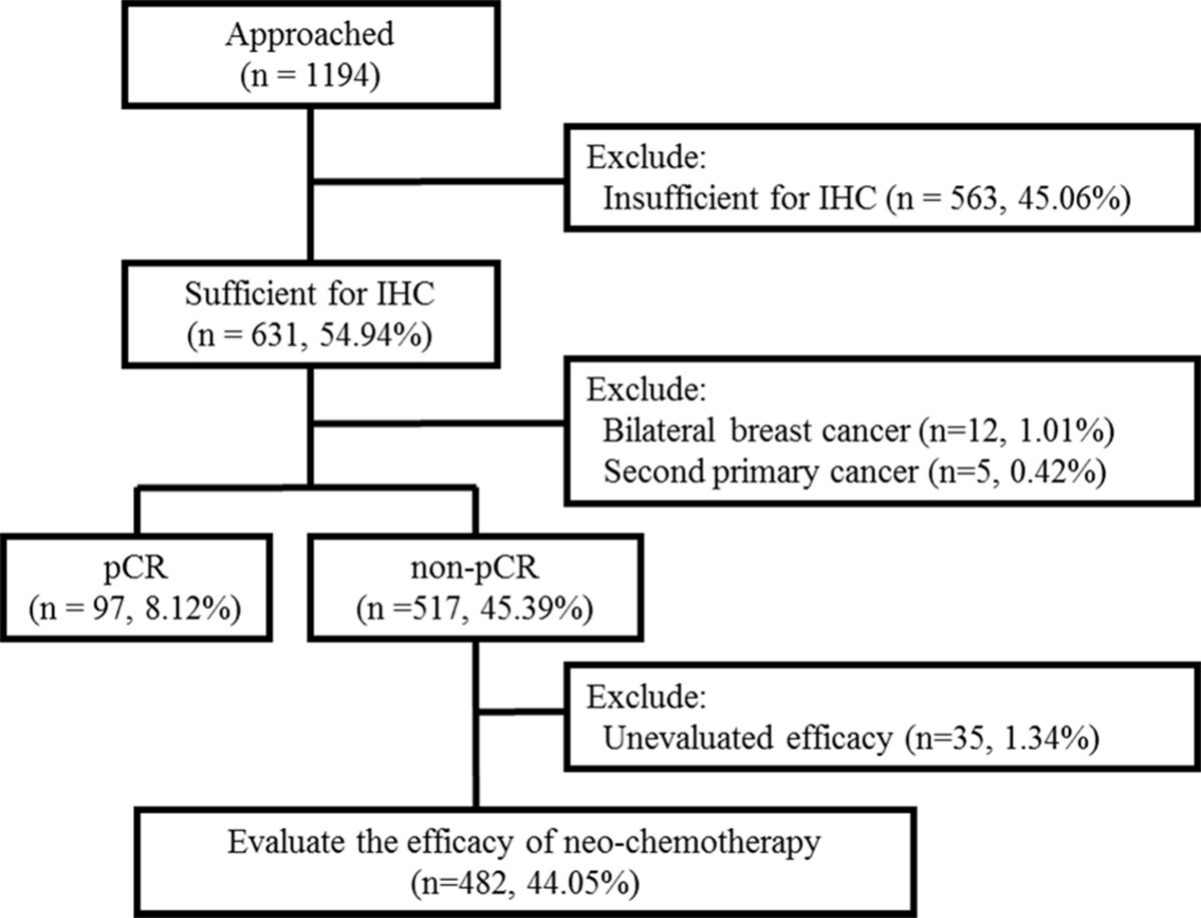
Flow diagram for the study.

We retrospectively collected data from patients with primary breast cancer who were treated with both NAC and subsequent surgery at the Zhejiang Cancer Hospital between January 2008 and December 2018. Although 1194 patients were initially identified for inclusion in the study, we excluded patients who were unevaluable for immunohistochemical (IHC) analyses, who had another primary cancer or bilateral primary breast cancer at the time of initial diagnosis. Patients who had a pathological complete response (pCR) after NAC were also excluded. Clinical stage was assessed according to the American Joint Committee on Cancer guidelines [9]. A flow diagram of patient selection is shown in Fig 1. Patients received at least four cycles of anthracycline- and/or paclitaxel-based NAC regimens. Trastuzumab was routinely recommended as targeted therapy for patients with human epidermal growth factor receptor 2 (HER2)-positive cancers.

We evaluated the concordance of HR expression throughout NAC by measuring ER, PR and Ki-67 expression with IHC in the preoperative core needle biopsy and in subsequent surgically resected specimens post-NAC. The proportion of positively stained tumor cells was used to define tumor ER and PR positivity, where HR-positive tumors were defined as having ≥1% stained tumor cells. Cancer cell proliferation was assessed by measuring Ki-67-positive tumor cells within the tissue section. The proliferation rate was defined based on the International Ki-67 in Breast Cancer Working Group [10], and was defined as the proportion of positive cells (at least 500–1000 cells) with nuclear staining at the invasive front of the tumor. Ki-67 expression >20% was considered high. After NAC, an increase in Ki-67 expression of >20% was considered an up-regulation, and a decrease of expression of >20% was considered down-regulation. Ki-67 changes < = 20% were considered stable. HER2 IHC and fluorescence in situ hybridization results were interpreted using College of American Pathologists/American Society of Clinical Oncology guidelines practiced at the time of diagnosis [11, 12]. Tumor subtypes were defined based on the expression of HR and HER2 as follows: Luminal (ER+ and/or PR+, HER2-), Luminal-HER2 (ER+ and/or PR+, HER2+), HER2-rich (ER- and PR-, HER2+), and triple-negative (ER- and PR- and HER2-).

The clinical response of breast and axillar tumors was assessed according to the Response Evaluation Criteria in Solid Tumors (RECIST) guidelines (version 1.1) [13]. The most appropriate method was utilized for measuring tumor response, and included sonography, mammography, magnetic resonance imaging and physical examination. Pathological complete response (pCR) was defined as having no remaining invasive disease in any excised breast tissue irrespective of nodal involvement. Clinical partial response (cPR) was defined as having a total reduction of target lesion diameter of ≥30%. Clinical progressive disease (cPD) was defined as having the total growth of target lesion diameter ≥20%. Clinical stable disease (cSD) was defined as having neither a cPR classification nor a cPD classification [14].

The expression changes in HR and Ki-67 were analyzed as categorical variables. A χ^2^ test was used to examine the association between HR and Ki-67 expression and the clinicopathological factors of the patients. The overall survival (OS) time of patients was defined as the time from diagnosis until the time of death. Disease-free survival (DFS) time was defined as the time between diagnosis and relapse. Patients who were alive at the end of the study (December 31, 2018) were referred to as censored observations. A Kaplan-Meier analysis was performed to investigate the disease-free survival (DFS) and overall survival (OS) of patients and a log-rank test was conducted to determine significant differences. Patient data parameters with *p*<0.1 in univariate analyses were used for multivariate analyses. Forward conditional logistic regression analyses were also performed. *p*<0.05 was considered statistically significant. All statistical analyses were performed using SPSS software version 24 (IBM Corp.).

This study was approved by the Administration Ethics Committee of Zhejiang Cancer Hospital (IRB-2019-2 [wz]) and conducted in accordance with the Principles of Helsinki Declaration. Written informed consent was obtained for each patient, and all data were fully anonymized before been accessed.

## Results

### Patient demographics and baseline characteristics

Overall, 482 non-pCR female patients with primary breast cancer who were treated with NAC at the Zhejiang Cancer Hospital from January 2008 to December 2018 were included in this retrospective study. The demographic and clinicopathologic characteristics of the participants are summarized in Table 1. The median age of enrolled patients was 50±9.2 years (range 21–75 years), with 16.8% of patients being 40 years old or younger, and 45.6% of patients being premenopausal. Clinical tumor stage, nodal stage, clinical stage, histologic type and histologic grade were predominantly cT2 (313, 64.9%), cN1 (283, 58.7%), stage II (285, 59.1%), ductal type (452, 93.8%), and grade 2 (137, 28.4%), respectively. All patients were treated with anthracycline- or taxane-based NAC regimens. A total of 336 (69.7%) patients had a clinical response (cPR) to NAC based on RECIST criteria. Moreover, 146 (30.3%) patients had no response, including 137 (28.4%) patients with cSD and nine (1.9%) patients with cPD. Within this study, 46.5% of patients had Luminal subtype tumors, 16.6% of patients had Luminal-HER2 tumors, 15.6% had HER2-rich tumors, and 17.2% had triple negative breast cancer.

**Table 1:** The relationship between pathological characteristics and discordance of biomarkers. [N (%)].

Demographic or Clinical Characteristic	No. of Patients (N = 482,%)	Estrogen Receptor			Progesterone Receptor			Ki-67			
		Concordant (n = 432)	Discordanc e (n = 50)	*P*	Concordant (n = 400)	Discordanc e (n = 82)	*P*	Declined (n = 243)	Concordan t (n = 75)	Increased (n = 130)	*P*
Age, years											
≤40	81(16.8)	68(84.0%)	13(16.0%)	0.066	60(74.1%)	21(25.9%)	**0.019**	33(43.4%)	18(23.7%)	25(32.9%)	0.079
>40	401(83.2)	364(90.8%)	37(9.2%)		340(84.8%)	61(15.2%)		210(56.5%)	57(15.3%)	105(28.2%)	
Menopausal											
Premenopaus	262(54.4)	233(88.9%)	29(11.1%)	0.585	212(80.9%)	50(19.1%)	0.187	131(54.6%)	39(16.3%)	70(29.2%)	0.956
Postmenopau	220(45.6)	199(90.5%)	21(9.5%)		188(85.5%)	32(14.5%)		112(53.8%)	36(17.3%)	60(28.8%)	
T Stage											
T1/0	41(8.5)	37(90.2%)	4(9.8%)	0.817	35(85.4%)	6(14.6%)	0.977	19(52.8%)	9(25.0%)	8(22.2%)	0.450
T2	313(64.9)	283(90.4%)	30(9.6%)		259(82.7%)	54(17.3%)		164(56.0%)	43(14.7%)	86(29.4%)	
T3	68(14.1)	60(88.2%)	8(11.8%)		56(82.4%)	12(17.6%)		30(47.6%)	15(23.8%)	18(28.6%)	
T4	60(12.5)	52(86.7%)	8(13.3%)		50(83.3%)	10(16.7%)		30(53.6%)	8(14.3%)	18(32.1%)	
N Stage											
N0	97(20.1)	90(92.8%)	7(7.2%)	0.336	85(87.6%)	12(12.4%)	0.435	49(54.4%)	14(15.6%)	27(30.0%)	**0.037**
N1	283(58.7)	253(89.4%)	30(10.6%)		232(82.0%)	51(18.0%)		143(54.8%)	35(13.4%)	83(31.8%)	
N2	55(11.4)	50(90.9%)	5(9.1%)		43(78.2%)	12(21.8%)		26(50.0%)	12(23.1%)	14(26.9%)	
N3	47(9.8)	39(83.0%)	8(17.0%)		40(85.1%)	7(14.9%)		25(55.6%)	14(31.1%)	6(13.3%)	
pT Stage											
pT1/0	153(31.7)	140(91.5%)	13(8.5%)	0.734	130((85.0%)	23(15.0%)	0.933	77(50.3%)	37(24.2%)	39(25.5%)	0.331
pT2	238(49.4)	212(89.1%)	26(10.9%)		198(83.2%)	40(16.8%)		119(50.0%)	35(14.7%)	84(35.3%)	
pT3	31(6.4)	28(90.3%)	3(9.7%)		25(80.6%)	6(19.4%)		14(45.2%)	7(22.6%)	10(32.3%)	
pT4	60(12.5)	52(86.7%)	8(13.3%)		50(83.3%)	10(16.7%)		31(51.7%)	9(15.0%)	20(33.3%)	
pN Stage											
pN0	102(21.2)	90(88.2%)	12(11.8%)	0.401	89(87.3%)	13(12.7%)	0.504	55(53.9%)	17(16.7%)	30(29.4%)	0.067
pN1	275(57.1)	223(81.1%)	52(18.9%)		220(80.0%)	55(20.0%)		148(53.8%)	23(8.4%)	104(37.8%)	
pN2	60(12.4)	55(91.7%)	5(8.3%)		53(88.3%)	7(11.7%)		34(56.7%)	16(26.7%)	10(16.7%)	
pN3	45(9.3)	39(86.7%)	6(13.3%)		38(84.4%)	7(15.6%)		24(53.3%)	14(31.1%)	7(15.6%)	
Histology											
Ductal	452(93.8)	404(89.4%)	48(10.6%)	0.757	372(82.3%)	80(17.7%)	0.119	228(54.2%)	69(16.4%)	124(29.5%)	0.612
Mixed	30(6.2)	28(93.3%)	2(6.7%)		28(93.3%)	2(6.7%)		15(55.6%)	6(22.2%)	6(22.2%)	
Nuclear Grade											
Ⅰ	11(2.3)	11(100.0%)	0(0.0%)	0.114	8(72.7%)	3(27.3%)	0.537	8(88.9%)	0(0.0%)	1(11.1%)	**< .001**
Ⅱ	137(28.4)	128(93.4%)	9(6.6%)		117(85.4%)	20(14.6%)		87(68.0%)	13(10.2%)	28(21.9%)	
Ⅲ	106(22.0)	92(86.8%)	14(13.2%)		89(84.0%)	17(16.0%)		44(43.1%)	33(32.4%)	25(24.5%)	
Unknow	228(47.3)										
Therapeutic Evaluation											
cCR/cPR	336(69.7)	298(88.7%)	38(11.3%)	0.216	273(81.3%)	63(18.8%)		161(52.3%)	51(16.6%)	96(31.2%)	
cSD	137(28.4)	127(92.7%)	10(7.3%)		120(87.6%)	17(12.4%)	0.229	79(60.3%)	20(15.3%)	32(24.4%)	0.102
cPD	9(1.9)	7(77.8%)	2(22.2%)		7(77.8%)	2(22.2%)		3(33.3%)	4(44.4%)	2(22.2%)	
Stage											
ⅡA	101(21.0)	94(93.1%)	7(6.9%)	0.234	86(85.1%)	15(14.9%)	0.752	53(57.6%)	12(13.0%)	27(29.3%)	0.393
ⅡB/ⅢA	282(58.5)	254(89.8%)	29(10.2%)		232(82.0%)	51(18.0%)		139(52.7%)	43(16.3%)	82(31.1%)	
ⅢB/ⅢC	99(20.5)	84(85.7%)	14(14.3%)		82(83.7%)	16(16.3%)		51(55.4%)	20(21.7%)	21(22.8%)	
Subtype											
Luminal	224(46.5)	208(92.9%)	16(7.1%)	**< .001**	180(80.4%)	44(19.6%)	**< .001**	128(62.1%)	19(9.2%)	59(28.6%)	**< .001**
Luminal/HER	80(16.6)	57(71.3%)	23(28.8%)		56(70.0%)	24(30.0%)		33(45.8%)	12(16.7%)	27(37.5%)	
HER2-rich	75(15.6)	68(90.7%)	7(9.3%)		70(93.3%)	5(6.7%)		36(50.7%)	16(22.5%)	19(26.8%)	
TNBC	83(17.2)	79(95.2%)	4(4.8%)		77(92.8%)	6(7.2%)		33(41.3%)	26(32.5%)	21(26.3%)	
Unknow	20(4.2%)										
NAC Regimens											
Anthracycline	38(7.9)	35(92.1%)	3(7.9%)	0.823	33(86.8%)	5(13.2%)	0.073	23(65.7%)	4(11.4%)	8(22.9%)	0.313
Taxane based	64(13.3)	58(90.5%)	6(9.5%)		59(92.2%)	5(7.8%)		37(62.7%)	7(11.9%)	15(25.4%)	
E+T both	380(78.8)	339(89.2%)	41(10.8%)		308(81.1%)	72(18.9%)		183(51.7%)	64(18.1%)	107(30.2%)	

Abbreviations: NAC, neoadjuvant chemotherapy; TNBC, triple negative breast cancer; HER-2, human epidermal growth factor receptor 2; cPR, clinical partial response; cSD, clinical stable disease; cPD, clinical progressive disease; A, Anthracycline; P, paclitaxel.

### Discordance in hormone receptor and Ki-67 expression throughout treatment

**Fig 2:**
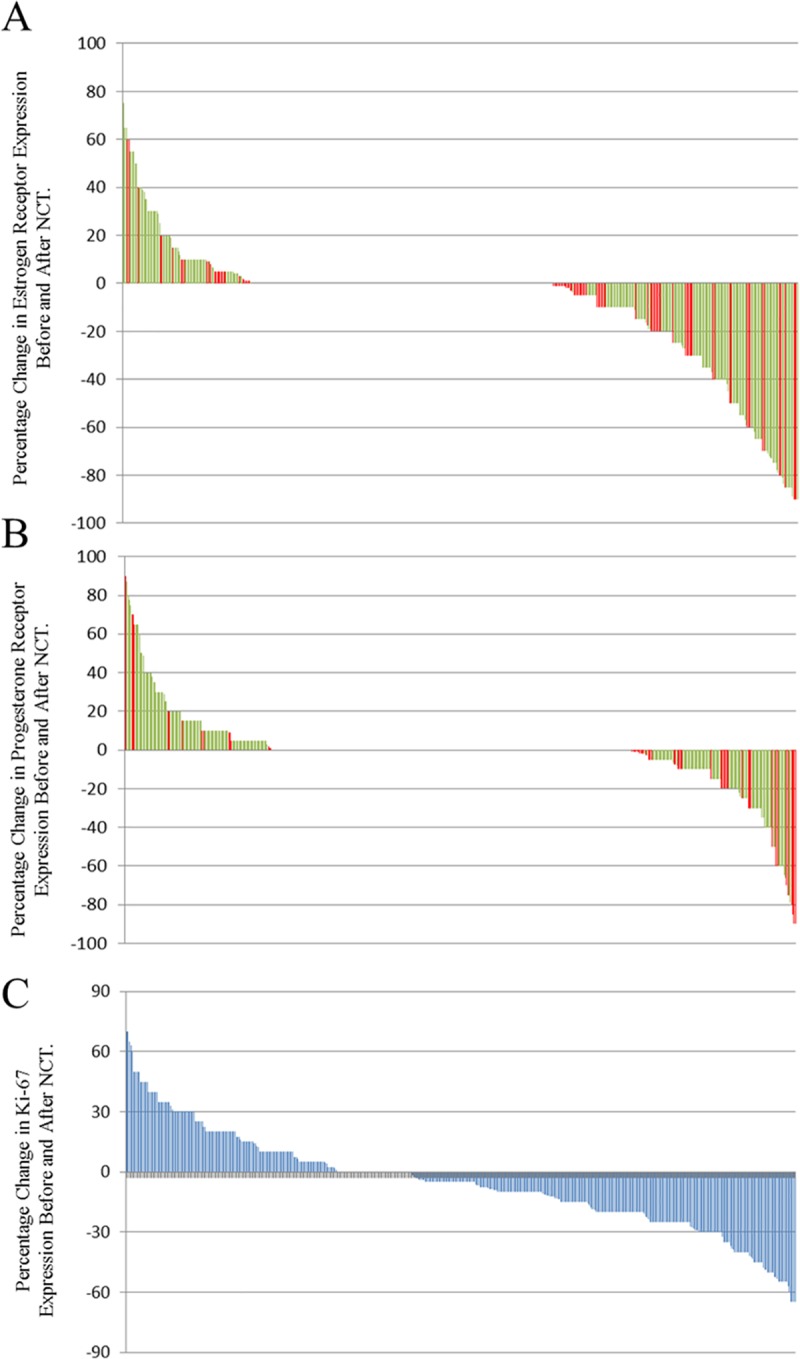
Waterfall plot showing the quantitative changes in ER, PR, and Ki-67 expression. Positive scores represent an increase in receptor expression before and after NAC; negative scores represent a decrease in receptor expression. Green, concordance with the primary tumor; Red, discordance with the primary tumor. (A) ER discordance rate: 10.4% (50/482) of patients, including 36 (7.5%) patients with positive-to-negative conversion and 14 (2.9%) patients with negative-to-positive conversion; (B) PR discordance rate: 17.0% (82/482) of patients, including 57 (11.8%) patients with positive-to-negative conversion and 25 (5.2%) patients with negative-to-positive conversion; (C) Ki-67 discordance rates: 50.4% (243/448) of patients had decreased expression and 27.0% (130/448) of patients had increased expression.

IHC data revealed three main groups of HR staining expression when comparing pre- and post-NAC: negative-to-positive conversion, positive-to-negative conversion and concordant. Ki-67 expression changes were divided into three groups: decreased expression, increased expression and stable expression. Quantitative changes in HR and Ki-67 expression are shown in Fig 2. ER and PR positive rates in core needle biopsies and excised tissue biopsies were 62.9% and 58.3%, and 55.8% and 49.2%, respectively. Discordance in ER and PR expression pre- and post-NAC was found in 50 (10.4%) and 82 (17.0%) patients, respectively, including 36 (7.5%) patients with ER positive-to-negative conversion, 14 (2.9%) cases with ER negative-to-positive conversion, 25 (5.2%) cases with PR negative-to-positive conversion and 57 (11.8%) cases with PR positive-to-negative conversion. These findings are similar to those reported in large studies for receptor status [8, 15]. A total of 448 patients were analyzed for Ki-67 status changes before and after NAC. A decrease in Ki-67 expression after NAC was found in 243 (50.4%) patients, while 130 (27.0%) patients had an increase in Ki-67 expression after NAC.

A Wilcoxon test was utilized to assess the relationship between pathological characteristics and biomarker expression discordance. Patients with Luminal-HER2 tumors were more likely to have inconsistent HR expression after NAC (Table 1). In comparison, changes in Ki-67 expression were correlated with several factors. Patients with N_0-1_, Grade I, and the Luminal tumor subtype were more likely to have a decrease in Ki-67 expression after NAC.

### Survival analysis

Follow-up data was available for all 482 patients. The median follow-up time was 49.24 months (range: 10.37–93.77 months).

**Fig 3:**
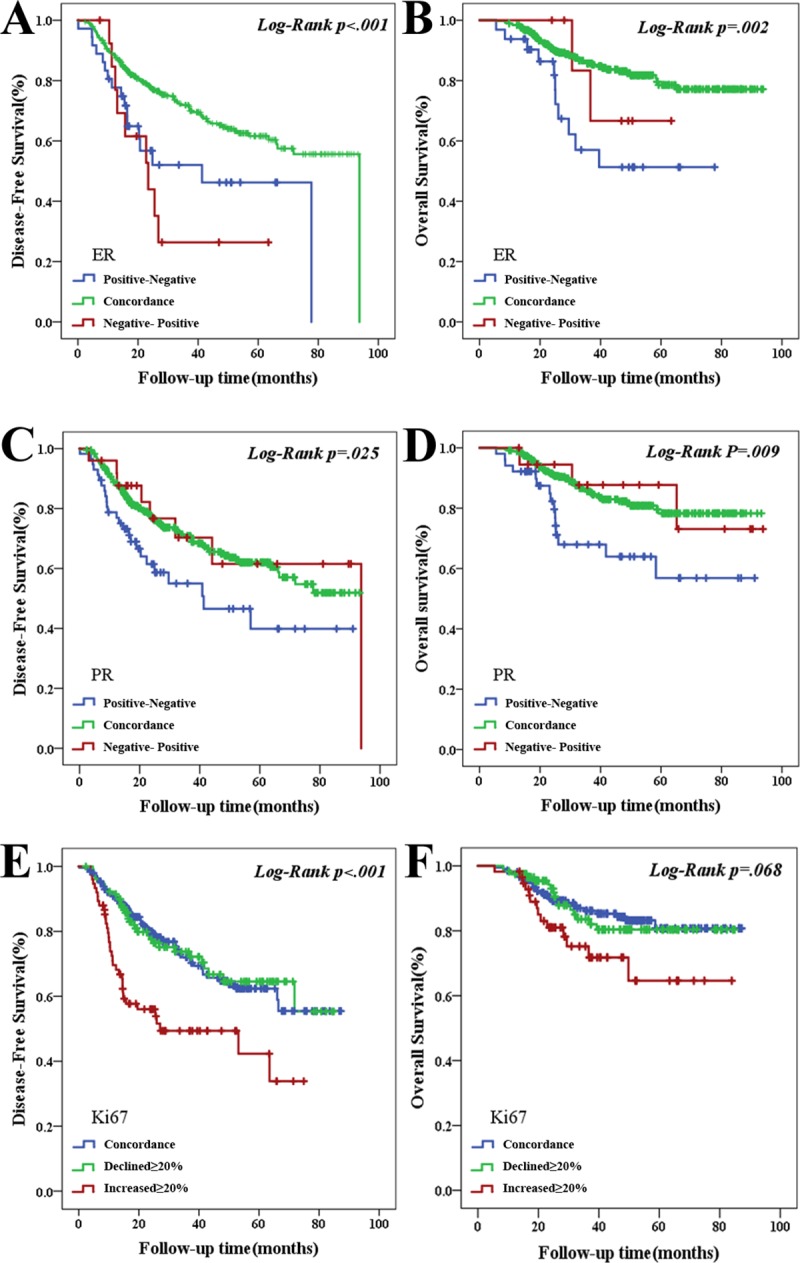
Kaplan-Meier curve of survival in patients with ER, PR and Ki-67 discordance. (A) DFS for ER discordance, p < .001; (B) OS for ER discordance, p = .002; (C) DFS for PR discordance, p = .025; (D) OS for PR discordance, p = .009. (E) DFS for Ki-67 discordance, p < .001; (F) OS for Ki-67 discordance, p = .068.

The HR positive-to-negative conversion patients had a significantly worse prognosis when compared with other groups (Fig 3A–3D). The median OS was 79.90±1.72, 53.47±5.78 and 51.83±5.79 months for the ER concordant, negative-to-positive and positive-to-negative conversion patients, respectively (χ2 = 12.08, *p* = 0.002). The median DFS was 67.14±8.76, 49.68±5.84 and 64.04±2.19 months for the PR negative-to-positive, positive-to-negative and concordant patients, respectively (χ2 = 7.37, *p* = 0.025). The median OS was 79.73±1.76, 80.82±6.62 and 63.88±5.60 months for the three PR groups (χ2 = 9.52, *p* = 0.009). The patients with Ki-67 expression that increased by ≥20% had a worse DFS when compared with stable or decreased Ki-67 expression (χ2 = 20.801, *p* <0.001, Fig 3E). However, there was no statistically significant difference in OS (χ2 = 5.38, *p* = 0.068, Fig 3F) between the Ki-67 expression groups. The prognosis of the stable Ki-67 expression group and the decreased-Ki-67 expression group was the same.

The loss of PR expression was associated with a significantly worse OS (Table 2). The risk of death in patients with PR positive-to-negative conversion was 6.58 times greater than that for patients with stable PR expression (hazard ratio = 6.58; 95% CI 2.03–21.37; *p* = 0.002). Ki-67 expression increases ≥20% were associated with a significantly worse DFS. The risk of disease recurrence in patients with increased Ki-67 expression was 1.91 times greater than for patients with stable Ki-67 expression (hazard ratio = 2.05; 95% CI 1.11–3.80; *p* = 0.02).

**Table 2:** Predictors of survival.

Factor	Univariate Analysis			Multivariate Analysis		
	Hazard Ratio	95%CI	*p*	Hazard Ratio	95%CI	*p*
**Overall survival**						
ER status						
Concordance	1.00	-	-	1.00	-	-
Negative to Positive	1.50	0.37 to 6.17	0.571	0.03	0 to 1.03	0.052
Positive to Negative	2.99	1.56 to 5.72	**< .001**	1.54	0.28 to 8.36	0.618
PR status						
Concordance	1.00	-	-	1.00	-	-
Negative to Positive	0.96	0.3 to 3.08	0.944	1.50	0.18 to 12.34	0.708
Positive to Negative	2.40	1.34 to 4.28	**0.003**	6.58	2.03 to 21.37	**0.002**
Ki-67 score						
Concordance	1.00	-	-	1.00	-	-
Declined≥20%	1.08	0.58 to 1.99	0.810	1.45	0.59 to 3.55	0.413
Increased≥20%	2.06	1.08 to 3.92	**0.028**	1.95	0.74 to 5.18	0.178
**Distant disease–free survival**						
ER status						
Concordance	1.00	-	-	1.00	-	-
Negative to Positive	2.87	1.46 to 5.67	**0.002**	0.56	0.14 to 2.21	0.408
Positive to Negative	2.00	1.21 to 3.33	**0.007**	1.17	0.35 to 3.92	0.801
PR status						
Concordance	1.00	-	-	1.00	-	-
Negative to Positive	0.88	0.41 to 1.89	0.750	2.47	0.71 to 8.6	0.155
Positive to Negative	1.78	1.16 to 2.74	**0.009**	2.04	0.87 to 4.76	0.100
Ki-67 score						
Concordance	1.00	-	-	1.00	-	-
Declined≥20%	0.98	0.65 to 1.48	0.942	1.10	0.58 to 2.12	0.767
Increased≥20%	2.35	1.57 to 3.54	**< .001**	1.91	1.02 to 3.58	**0.043**

Abbreviations: CI: confidence interval.

## Discussion

Currently, NAC has been one of the most effective adjuvant treatments for breast cancer patients who have inoperable cancer or who wish to have breast-conserving surgery. Several prior studies have reported the discordance of HR and Ki-67 expression pre- and post-NAC [14, 16, 17]. Van de Ven *et al*. indicated in a meta-analysis that the discordance in HR status pre- and post-treatment ranged from 8%–33% in patients who received NAC [17]. For ER and PR status, discordances of 2.5%–17% and 5.9%–51.7%, respectively, were separately reported [17]. Our study demonstrates the instability in biomarker expression throughout NAC. In the present study, 55.2% (266/482) of patients receiving NAC experience at least one kind of HR status alteration. For ER, PR, and Ki-67 expression changes, 10.4% (50/482), 17.0% (82/482), and 77.4% (373/448) of patients experienced discordance during NAC, respectively. These changes verify the presumption that the discordance in biomarker expression is elicited by NAC.

Several recent studies indicate that the failure to detect negative-to-positive expression changes in tumors is likely to have a greater impact on treatment decisions than the failure to detect positive-to-negative expression changes in tumors. If endocrine treatment is administered to patients with a negative-to-positive conversion, an improved OS and DFS is observed [18, 19]. However, we did not observe similar trends in our study. In contrast, we found that patients with HR-positive tumors that switched to an HR-negative status had a worse OS and DFS than patients whose tumors remained HR stable or exhibited a negative-to-positive conversion after NAC. This conclusion demonstrates from another perspective the essential role of endocrine treatment in patients with an HR negative-to-positive status conversion. These results support the necessity to evaluate biopsy specimens both before and after NAC. The pre- and post-NAC HR status would help determine the appropriate administration of adjuvant endocrine treatment. We believe that endocrine treatment can be administered in patients with HR-positive tumors at least once prior to or after NAC.

According to previous reports, there are several possible mechanisms explaining HR expression changes in breast cancer tumors after NAC. Chemotherapy could induce the change to a positive HR status since all tumor cells are originally derived from well-differentiated HR-positive breast cancer cells [17]. Another explanation would be the selection of tumor cell clones during treatment, with a selective disappearance of either HR-positive or HR-negative tumor cells. NAC could upregulate some proteins favoring the expression or re-expression of HR in the tumor nuclei. It is generally known that HR-negative tumors are more sensitive to chemotherapy than HR-positive tumors, and this theory is explained by the upregulation of HR [18]. Lastly, Huang *et al*. proposed a hypothesis that some HR-positive tumor cells may be more sensitive to chemotherapy. A “neo-endocrinochemotherapy” approach can thus be taken, where tumor cell sensitivity to chemotherapy could be enhanced by providing endocrine hormones to patients before and/or during chemotherapy [20].

The 2011 and 2013, the St. Gallen Consensus Conference recommended adding Ki-67 as a proliferation biomarker for breast cancer subtypes such as Luminal A and Luminal B [21, 22]. In our study, discordance in Ki-67 status pre- and post-NAC was observed in most patients. Similar to other studies [23], our research found that 50.4% of patients exhibited a significant decrease in Ki-67 expression. However, we also found that 27.0% of patients displayed a significant increase in Ki-67 after NAC. In previous studies, high Ki-67 expression post-treatment was one of the most important prognostic predictors for clinical outcome [14, 24, 25]. Ki-67 index was also a predictive biomarker for a pathologic complete response. Patients with low Ki-67 expression had a comparable outcome compared with patients with a pathologic complete response [26]. In the present study, it is worth noting that patients with high Ki-67 expression after NAC had a greater risk of shortened DFS and OS compared with the other cohorts. This result is consistent with Yoshioka’s research findings [16].

There are several possible mechanisms that can explain increased Ki-67 expression following NAC. Chemotherapy mostly kills tumor cells that actively proliferate, which indirectly promotes residual tumor cells in the G0 phase to metabolize actively or enter into the division cycle again. In this case, Ki-67 could be re-expressed in tumor cells. On the other hand, the link between high Ki-67 expression and the development of chemotherapy resistance could also explain the increased Ki-67 expression following NAC. Marcom *et al*. [27] and Oh *et al*. [28] revealed that increased expression of HER2-associated genes along with a cell proliferation signature that includes *MKI67*, *CCNB1*, and *MYBL2*, was associated with drug resistance, which further led to high Ki-67 expression.

This retrospective analysis has shortcomings and limitations. There were inconsistencies in the technical processes in the beginning of the study. Therefore, the IHC information from 563 patients was incomplete, and only 482 patients met the study conditions. Although improvements in radiology has resulted in increased accessibility of most tissue by minimally invasive methods, different way tumor shrink during NAC make it difficult to choose an appropriate puncture site in the residual tumor foci. Finally, because of the diversity of chemotherapy regimens, we did not have a stratified analysis for the number of chemotherapy cycles.

## Conclusions

In summary, the discordance rates of ER, PR and Ki-67 expression pre- and post-NAC were 10.4%, 17.0%, and 84.4%, respectively. The most common change observed was the loss of PR expression. Patients with an HR positive-to-negative conversion or increased expression of Ki-67 after NAC had a significantly worse prognosis. We validated the PR positive-to-negative conversion and increased Ki-67 expression as independent predictors of poor prognosis. This study shows that re-examination of biomarker expression should be conducted after NAC, which may result in altered treatment for about 17% of patients, as well as an opportunity to optimize adjuvant systemic therapy regimens and reassess prognosis.

## Supporting information

S1 Dataset (XLS)

## Abbreviations

ER: estrogen receptor
PR: progesterone receptor
HR: hormone receptor
DFS: disease-free survival
OS: overall survival
NAC: neoadjuvant chemotherapy
IHC: immunohistochemistry
pCR: pathological complete response
TNBC: triple negative breast cancer
HER2: human epidermal growth factor receptor 2
cPR: clinical partial response
cSD: clinical stable disease
cPD: clinical progressive disease
